# A Novel Piezo1 Agonist Promoting Mesenchymal Stem Cell Proliferation and Osteogenesis to Attenuate Disuse Osteoporosis

**DOI:** 10.1002/smsc.202400061

**Published:** 2024-06-30

**Authors:** Ruihan Hao, Hairong Tang, Chunyong Ding, Bhavana Rajbanshi, Yuhang Liu, Ding Ma, Zhouyi Duan, Yuxin Qi, Liming Dai, Bingjun Zhang, Ao Zhang, Xiaoling Zhang

**Affiliations:** ^1^ Department of Orthopedic Surgery Xin Hua Hospital Affiliated to Shanghai Jiao Tong University School of Medicine (SJTUSM) Shanghai 200092 China; ^2^ Shanghai Frontiers Science Center of Targeted Drugs School of Pharmaceutical Sciences Shanghai Jiao Tong University Shanghai 200240 China; ^3^ Department of Dermatology and Venereology Tongji University School of Medicine Shanghai 200092 China; ^4^ Collaborative Innovation Centre of Regenerative Medicine and Medical BioResource Development and Application Co‐constructed by the Province and Ministry Guangxi Medical University Nanning Guangxi 530021 China; ^5^ National Facility for Translational Medicine (Shanghai) Shanghai 200240 China

**Keywords:** disuse osteoporosis, mechenchymal stem cell proliferations, novel agonists, osteogenesis, Piezo1

## Abstract

Disuse osteoporosis (OP) is a state of bone loss due to lack of mechanical stimuli, probably induced by prolonged bed rest, neurological diseases, as well as microgravity. Currently the precise treatment strategies of disuse OP remain largely unexplored. Piezo1, a mechanosensitive calcium (Ca^2+^) ion channel, is a key force sensor mediating mechanotransduction and it is demonstrated to regulate bone homeostasis and osteogenesis in response to mechanical forces. Using structure‐based drug design, a novel small‐molecule Piezo1 agonist, MCB‐22‐174, which can effectively activate Piezo1 and initiate Ca^2+^ influx, is developed and is more potent than the canonical Piezo1 agonist, Yoda1. Moreover, MCB‐22‐174 is found as a safe Piezo1 agonist without any signs of serious toxicity. Mechanistically, Piezo1 activation promotes the proliferation of bone marrow mesenchymal stem cells by activating the Ca^2+^‐related extracellular signal‐related kinases and calcium–calmodulin (CaM)‐dependent protein kinase II (CaMKII) pathway. Importantly, MCB‐22‐174 could effectively promote osteogenesis and attenuate disuse OP in vivo. Overall, the findings provide a promising therapeutic strategy for disuse OP by chemical activation of Piezo1.

## Graphical abstract

A novel small‐molecule Piezo1 agonist, MCB‐22‐174, developed based on structure optimization, which could effectively activate Piezo1 and initiate Ca^2+^ influx, is more potent than the canonical Piezo1 agonist, Yoda1. MCB‐22‐174 could promote mesenchymal stem cell proliferation and osteogenesis to attenuate disuse osteoporosis in vivo effectively. The findings provide a drug candidate for disuse osteoporosis by chemical activation of Piezo1.

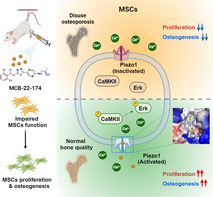

## 1 Introduction

Disuse osteoporosis (OP) describes a state of bone loss due to local skeletal unloading or systemic immobilization. Clinical settings of disuse OP include spinal cord injury, other neurological and neuromuscular disorders, immobilization after fractures and bed rest, as well as microgravity.^[^ ^1^ ^]^ The treatment of disuse OP by combining antiresorption drugs such as denosumab or bone anabolic drugs with physical exercise has shown positive, but sometimes inconsistent, results.^[^ ^1^, ^2^ ^]^ These indicate it is an emerging and important area of research that requires good understanding of both bone physiology and mechanics and developing new target therapeutic options for disuse OP. Previous studies have reported mesenchymal stem cells (MSCs) to be sensitive to mechanical stimulation by upregulating proliferation and osteogenic activity.^[^ ^3^, ^4^ ^]^ During mechanical loading, the osteogenic ability of osteoblast is enhanced, which improves bone quality.^[^ ^5^, ^6^ ^]^ Tatsumi et al. developed a transgenic mouse model with the ablation of osteocytes through the injection of diphtheria toxin.^[^ ^7^ ^]^ Importantly, these mice did not develop unloading‐induced bone loss, underlining the osteocyte's function in sensing mechanical loading and the development of disuse OP. These findings showed the importance of mechanical stimulation sensing of MSCs, osteoblasts, and osteocytes in bone mass maintenance.

A variety of cell surface structures and molecules are associated with mechanical stimulation sensing in bone cells such as integrins, focal adhesions, primary cilia, and ion channels.^[^ ^8^ ^]^ Among these structures, Piezo ion channels is important in varied cell types and Ardem Patapoutian received the 2021 Nobel Prize in Physiology or Medicine (jointly) for its recognition.^[^ ^9^ ^]^ Piezo1 is highly expressed in osteoblastic lineage and is assumed to be essential for osteogenesis, whereas knockout of Piezo1 led to severe bone loss and attenuated osteogenesis of osteoblast.^[^ ^10^, ^11^ ^]^ Piezo1 has already been proved to be able to be activated by chemical molecules, Yoda1, rendering the pharmacological activation of Piezo1 with small molecules a novel and effective strategy for disuse OP.^[^ ^12^ ^]^ Nevertheless, the development of small‐molecule Piezo1 agonist is still in its infancy with limited biochemical potency. Highly cellular potent or in vivo efficacious Piezo1 agonists are rarely available yet, and no drug‐like small molecules have entered clinical trials for proof‐of‐concept studies.

Thus, more efforts are urgently needed to provide developable Piezo1 agonists for preclinical and clinical validation studies. Herein, we developed a high‐activity specific agonist of Piezo1, MCB‐22‐174, which promotes osteoblastic differentiation better than Yoda1 both in vitro and in vivo and effectively attenuates hind‐limb unloading (HU) induced bone loss. Mechanistically, MCB‐22‐174‐activated Piezo1 promotes the proliferation of bone marrow MSCs by activating the CaMKII/extracellular signal‐related kinases (ERK) pathway. Overall, our efforts led to a promising chemical probe for physiological functional studies of Piezo1, as well as a drug candidate for further investigation.

## 2 Results

### 2.1 Piezo1 is a Significant Mechanotransductor of MSC, Regulating MSC Proliferation and Osteogenesis as Well as Bone Mass

**Figure 1:**
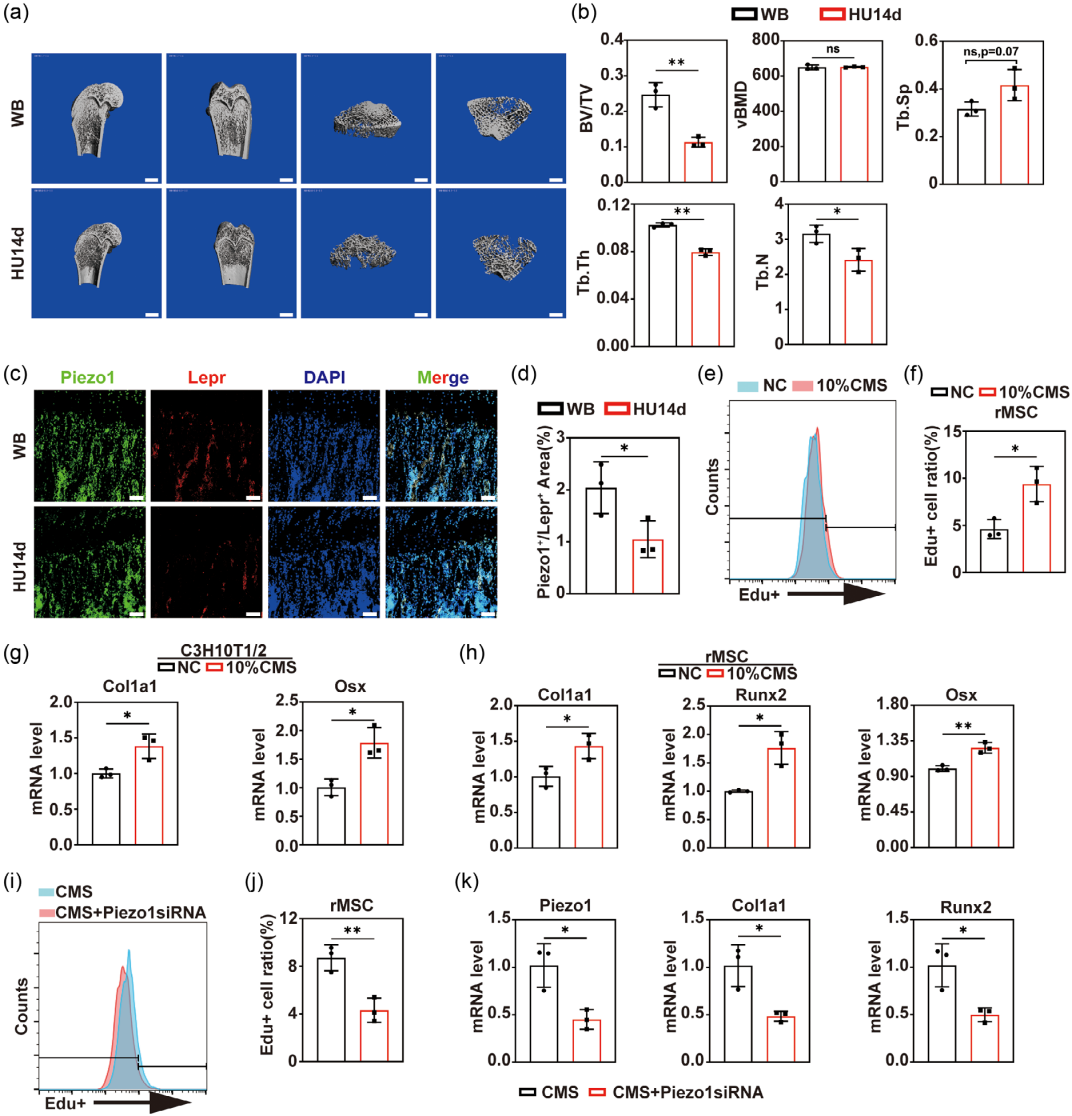
Piezo1 regulates MSC proliferation and osteogenesis as well as bone mass. a. μCT of rat distal femur from control and HU group. The left two lists of pictures are sagittal and coronal reconstruction images of the distal femur. The right two lists of pictures are the magnified trabecular bone structure from the most proximal growth plate of distal femur to the 200 slices underneath it. Scale bars equal to 2 mm. b) Statistic data of μCT (*n* = 3). BV/TV, bone volume/total volume. vBMD, volumetric bone mineral density. Tb.Th, trabecular thickness. Tb.Sp, trabecular separation. Tb.N. trabecular number. c) Immunofluorescent staining of Piezo1 and Lepr in the proximal right tibia of WB and HU14d groups (*n* = 3). Nuclei were counterstained with DAPI. Scale bars equal to 100 μm. d) Statistical analysis of costaining area in immunofluorescent staining (*n* = 3). e) Representative images of flow cytometry analysis of EdU+ rMSCs in control and 10% CMS groups. f) Statistical data of flow cytometry (*n* = 3). g) Relative expression of Col1a1 and Osx of C3H10T1/2 cell line in control and 10% CMS applicated groups h) Relative expression of Piezo1, Col1a1, Runx2, and Osx of rMSCs in control and 10% CMS applicated groups. i) Representative images of flow cytometry analysis of EdU+ rMSCs in control and Piezo1 siRNA transfected groups with 10% CMS application. j) Statistical data of flow cytometry (*n* = 3). k) Relative expression of Piezo1, Col1a1, Runx2, and Osx of rMSCs in control and Piezo1 siRNA transfected groups with 10% CMS. ns. No significance, *:*p* < 0.05, **:*p* < 0.01.

Piezo1 has been reported as a significant mechanotransductor in many cells and tissues, and it is essential in the bone anabolism process.^[^ ^11^, ^13^ ^]^ To explore the relationship between Piezo1 and disuse OP, we employed the HU model as a canonical model of disuse OP. In this model, the hind limbs of rats were detached from the ground by tail suspension to stimulate weightlessness (Figure S1A, Supporting Information).^[^ ^14^ ^]^ After 14 days of HU, rats were sacrificed and the quality of right distal femurs was measured by microcomputed tomography (μCT, **Figure** **1**a). We found a significant decline in bone volume ratio (BV/TV), thickness, and the number of trabecular bone (Tb.Th. and Tb.N), along with a rise of trabecular separation (Tb.Sp.) after HU (Figure 1b). However, volumetric bone mineral density (vBMD) measured by μCT showed no changes. Some previous studies indicated that the expression of Piezo1 decreased after in vitro microgravity treatment.^[^ ^11^ ^]^ However, the influence on in vivo Piezo1‐expressing MSCs due to loss of mechanical stimulation remains unclear. Thus, we conducted the immunofluorescent costaining of Piezo1 and Lepr (a marker of MSCs) in the proximal tibia. We found a significant decrease of Piezo1+/Lepr+ area in bone marrow after HU for 14 days (Figure 1c,d), demonstrating a decrease of Piezo1‐expressing MSCs in disuse OP.

To further clarify the role of Piezo1 in the proliferation and osteogenesis of MSC stimulated by mechanical stress, we generated an in vitro cyclic mechanical stretch (CMS) model. It resulted in an increase in EdU^+^ cell number in the wells after 10% CMS stimulation for 2 days with more newly generated cells and improved proliferation of rMSCs (Figure 1e,f). We also examined osteogenic properties in both primary rMSCs and C3H10T1/2 cell line (MSC cell line of mice) and found collagen I (Col1a1) and osterix (Osx) to be upregulated after 72 h of 10% CMS stimulation in C3H10T1/2 cell line (Figure 1g). As for primary rMSCs, osteogenic markers including Col1a1, Osx, and RUNX family transcription factor 2 (Runx2) were upregulated after 72 h of 10% CMS stimulation (Figure 1h). These results, including mechanical stimulation promoting MSC osteogenesis and proliferation, are similar to those reported previously, confirming our success in construction of CMS model.

Next, we investigated the significance of Piezo1 in mechanical signaling transduction. We knocked down Piezo1 in rMSCs by small interference RNA (siRNA) with efficiency reaching 70% at RNA level (Figure S1b, Supporting Information). Then, control group and Piezo1 knockdown rMSCs were treated by 10% CMS for 2 days to test the proliferation properties and 3 days to test osteogenesis properties. After CMS stimulation, the EdU^+^ cell ratio of Piezo1 knockdown group became less, compared to control groups (Figure 1i,j). Also, the osteogenic marker genes including Col1a1, Osx, and Runx2 were downregulated after Piezo1 knockdown under CMS stimulation (Figure 1k). These results demonstrated that Piezo1 played an essential role in mechanical signaling transduction.

### 2.2 Development and In Vitro Validation of a Novel Piezo1 Agonist

**Figure 2:**
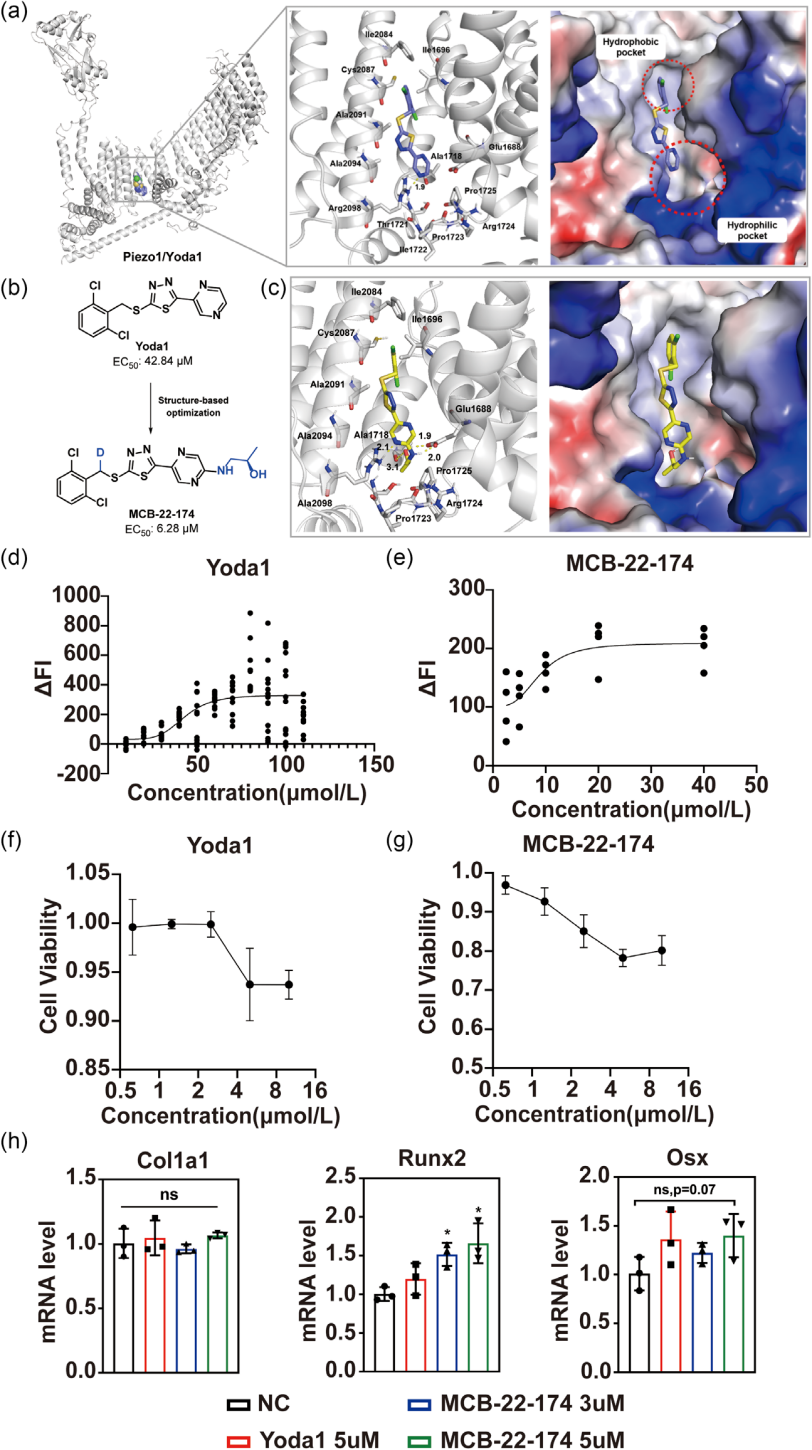
Development of Piezo1 agonist (MCB‐22‐174). a) The putative Yoda1 binding site in Piezo1 (PDB ID: 6B3R). Yoda1 is displayed in blue sticks, residues are depicted in white sticks. The allosteric binding pocket surrounded by residues Glu1688, Leu1689, Ala1718, Ala2091, Ala2094, Try2095, and Arg2098. b) Structure‐guided optimization of Yoda1. c) Docking mode of MCB‐22‐174 with Piezo1. Close‐up views of the hydrogen bonds and hydrophobic contacts formed between MCB‐22‐174 (yellow sticks) and Piezo1. Hydrogen bonds are shown in yellow dashed lines, and corresponding distances were labeled in angstrom. Interacting residues of Piezo1 are shown in the white stick representation. d) EC_50_ curve of Yoda1 in C3H10T1/2 cells. EC_50_ of Yoda1 equals to 42.84 μmol L^−1^ in C3H10T1/2 cell line. e) EC_50_ curve of MCB‐22‐174 in C3H10T1/2 cells. EC_50_ of MCB‐22‐174 equals 6.28 μmol L^−1^ in C3H10T1/2 cell line. f) Cell viability curve of Yoda1. g) Cell viability curve of MCB‐22‐174. h) Relative expression of Col1a1, Runx2, and Osx of rMSCs in control, 5 μmol L^−1^ Yoda1, 3, and 5 μmol·L^−1^ MCB‐22‐174‐treated groups for 72 h. (*n* = 3) ns. No significance, *:*p* < 0.05, compared with NC group.

Syeda et al.^[^ ^12^ ^]^ screened ≈3.25 million compounds and identified the first synthetic small‐molecule agonist targeting the Piezo1 channel, Yoda1 (**Figure** **2**a), which specifically but modestly activated both mouse and human Piezo1. Later, Botello‐Smith et al.^[^ ^15^ ^]^ identified an allosteric binding pocket of Yoda1 surrounded by residues Glu1688, Leu1689, Ala1718, Ala2091, Arg2098, and so on (Figure 2a), which is ≈40 Å away from the central ion‐conducting pore of Piezo1. From the docking analysis, there is ample space around Yoda1 unoccupied, especially around the pyrazine component, thus paving the way for rational design of more potent Piezo1 allosteric agonists (Figure 2a).^[^ ^16^ ^]^ To this end, we extended the pyrazine ring to a deeper pocket that could be fully occupied by a fitting group. Synthesis of these agonists involves introducing an amine to pyrazine through a nucleophilic substitution reaction (Figure S2a, Supporting Information) and its structure was further confirmed by ^1^H NMR and ^13^C NMR (Figure S2b,c, Supporting Information). MCB‐22‐174 was found to exhibit potent agonistic activity on Piezo1, with an EC_50_ value of 6.28 μmol L^−1^, which was eightfold more potent than that of Yoda1 (EC_50_: 42.84 μmol L^−1^, Figure 2d,e). Although the cellular toxicity of MCB‐22‐174 is slightly stronger than Yoda1, the cell viabilities of MSCs still reached over 80% at 16 μmol L^−1^, which is ≈3 times of the EC_50_ value of MCB‐22‐174 (Figure 2f,g), indicating MCB‐22‐174 has a reasonable safety index. As expected in Figure 2c, MCB‐22‐174 could neatly fit in the allosteric binding pocket of Piezo1, and the aminopropanol moiety well extended into the deeper pocket surrounded by residues Glu1688, Ala1718, Pro1723, Arg1724, and Pro1725. Moreover, three hydrogen bonds formed between the aminopropanol moiety and Glu1688 and Ala1718 have been identified. These binding interactions might contribute to the substantial increase in potency of MCB‐22‐174.

Based on our existing results, Piezo1 activation and Ca^2+^ influx is predicted to have a positive influence on MSCs osteogenesis. Thus, MCB‐22‐174‐treated MSCs resembled the phenotypes similar to mechanical stimulated ones. To confirm this assumption, we treated C3H10T1/2 cell line with 5 μmol L^−1^ Yoda1,3 μmol L^−1^, and 5 μmol L^−1^ MCB‐22‐174 for three days. Then, we detected osteogenic markers including Col1a1, Runx2, and Osx of treated cells by reverse transcription‐quantitive real‐time PCR (RT‐qPCR) . Results showed a significant rise of Runx2 mRNA level in 5 μmol L^−1^ MCB‐22‐174 treated group, along with a nonsignificant upregulation of Osx (*p* = 0.07, Figure 2h), indicating a better promotion of osteogenesis in MCB‐22‐174‐treated group. Also, MSCs have the potency of chondrogenesis and adipogenesis. We further examined the effect of 5 μmol L^−1^ Yoda1 and 5 μmol L^−1^ MCB‐22‐174, along with Piezo1 knockdown by siRNA, on chondrogenesis and adipogenesis of MSCs. Results showed a significant decline of chondrogenesis marker Comp expression in both Yoda1 and MCB‐22‐174 group (Figure S3a, Supporting Information). In addition, MCB‐22‐174 group showed a further decrease of chondrogenesis marker Acan, adipogenesis marker Lpl, and Fabp4 expression, indicating a more significant suppression of chondrogenesis and adipogenesis than Yoda1 (Figure S3a,b, Supporting Information).

### 2.3 Piezo1‐Mediated Mechanical Signaling Promotes MSC Proliferation via ERK and CaMKII‐Related Pathway

**Figure 3:**
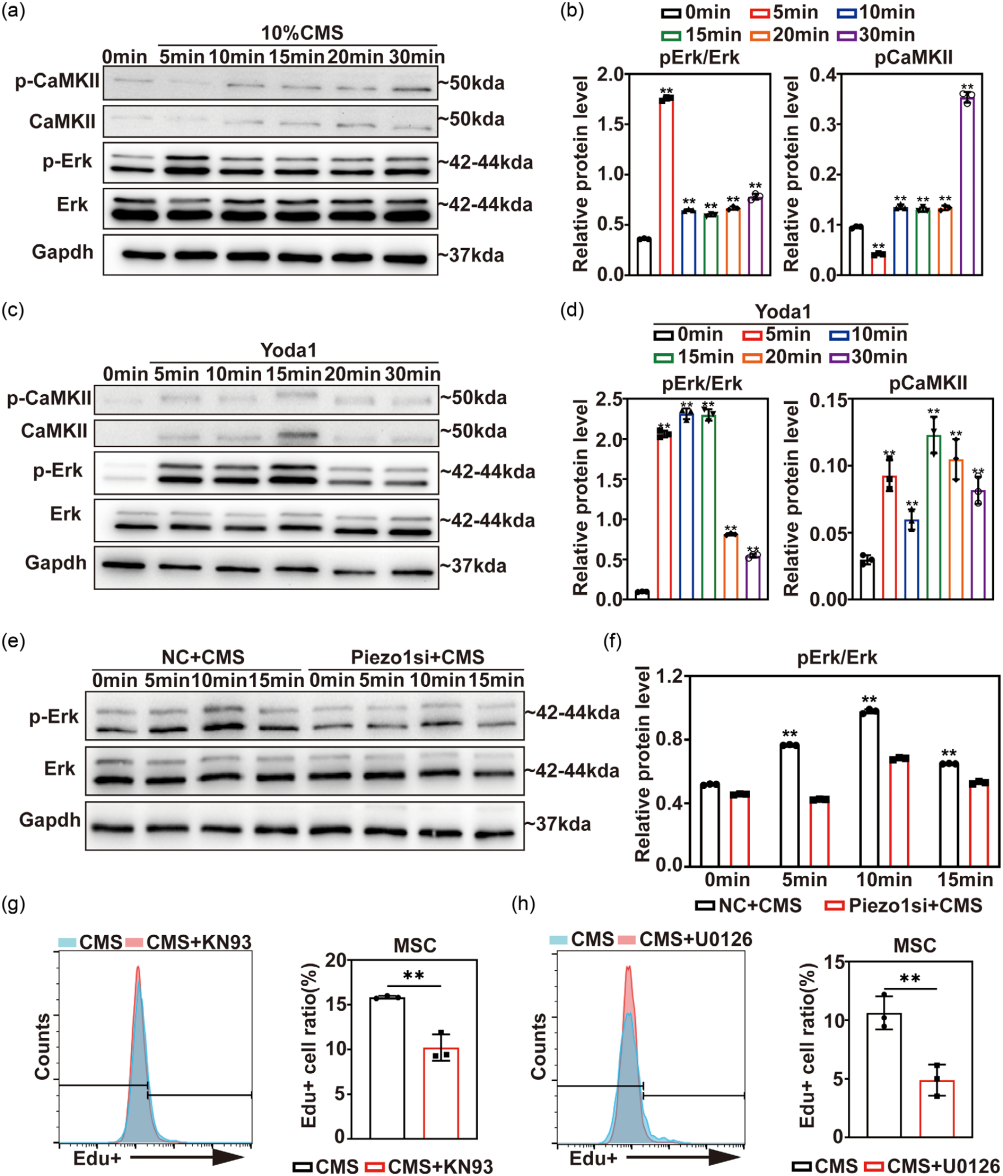
Piezo1 promoted the MSC proliferation through the CaMKII and ErK signaling pathway. a) Western blot images of CaMKII, p‐CaMKII, ERK, and p‐ERK in rMSCs after 0–60 min of 10% CMS stimulation. b) Statistical data of p‐ERK/ERK ratio and p‐CaMKII relative expression. c) Western blot images of CaMKII, p‐CaMKII, ERK, and p‐Eek in rMSCs after 0–60 min 5 μm Yoda1 treatment. d) Statistical data of p‐Erk/Erk ratio and *p*‐CaMKII relative expression. e) Western blot images of ERK and *p*‐Erk in control and Piezo1 siRNA transfected rMSCs after 0–15 min of 10% CMS treatment. f) Statistical data of p‐Erk/Erk ratio. g) Representative images and statistical data of flow cytometry analysis of EdU+ rMSCs in control and U1026‐treated groups. h) Representative images and statistical data of flow cytometry analysis of EdU+ rMSCs in control and KN93‐treated groups. For Western blot, GAPDH was employed as an internal reference (*n* = 3). *:*p* < 0.05, **:*p* < 0.01.

Mechanical signaling has been reported in literature to result in Piezo1 activation.^[^ ^17^ ^]^ Piezo1 is a kind of calcium ion channel and its activation initiates Ca^2+^ influx. To investigate the underlying mechanism of Piezo1 mediated MSC proliferation, we focused on intracellular Ca^2+^‐related mechanisms. The mitogen‐activated protein kinases (MAPK) and downstream ERK signaling pathway are calcium related, and the activation of MAPK/ERK pathway is reported to be associated with cell functions such as proliferation, differentiation, migration in tumor cells, and normal cell lines.^[^ ^18^, ^19^ ^]^ Calcium–calmodulin (CaM)‐dependent protein kinase II (CaMKII) pathway is closely related to intracellular calcium level, mediating cell proliferation in various cells.^[^ ^20^, ^21^ ^]^ Based on previous findings, we speculated an impact of the signaling pathways in rMSCs. We tested the expression of activation of ERK and CaMKII signaling pathway after 0–60 min of CMS stimulation by Western blot. Results showed more phosphate‐ERK (p‐ERK) and phosphate‐CaMKII (p‐CaMKII) were generated after CMS stimulation in rMSCs (**Figure** **3**a,b), indicating the stimulated activation of ERK and CaMKII pathway. Then, we applied a Piezo1 specific agonist, Yoda1, to activate Piezo1 without adding mechanical stimulation, which showed p‐ERK and p‐CaMKII were upregulated after 5–60 min of Yoda1 treatment (Figure 3c,d). The results indicated a relationship between mechanical stimulation, Piezo1 activation, and ERK/CaMKII signaling pathway activation. To further verify the relationship between signaling pathways and Piezo1, we knocked down Piezo1 by siRNA and stimulated the cells for 0–15 min with 10% CMS. Results showed a decline in the ratio of p‐ERK/ERK in Piezo1 siRNA transfected group, representing a decrease in activation of ERK signaling pathway after Piezo1 knockdown (Figure 3e,f), and confirmed MAPK/ERK signaling pathway to be closely related to Piezo1.

To further investigate the effect of the ERK signaling pathway and CaMKII signaling pathway on MSC proliferation, we used U1026 (inhibitor of ERK) and KN93 (inhibitor of CaMKII) to pretreat rMSCs and stimulated the cells by 10% CMS for 2 days. EdU staining showed a decline of EdU^+^ cell ratio after U1026 or KN93 treatment (Figure 3g,h), suggesting a reduction of the proliferation rate of MSCs after ERK or CaMKII inhibitor treatment. These results suggest that mechanical stimulation‐activated ERK and CAMKII signaling pathways are related to MSC proliferation.

### 2.4 MCB‐22‐174 More Efficiently Activates the CaMKII/Erk Signaling Pathway in Vitro via Chemically Induced Activation of Piezo1

**Figure 4:**
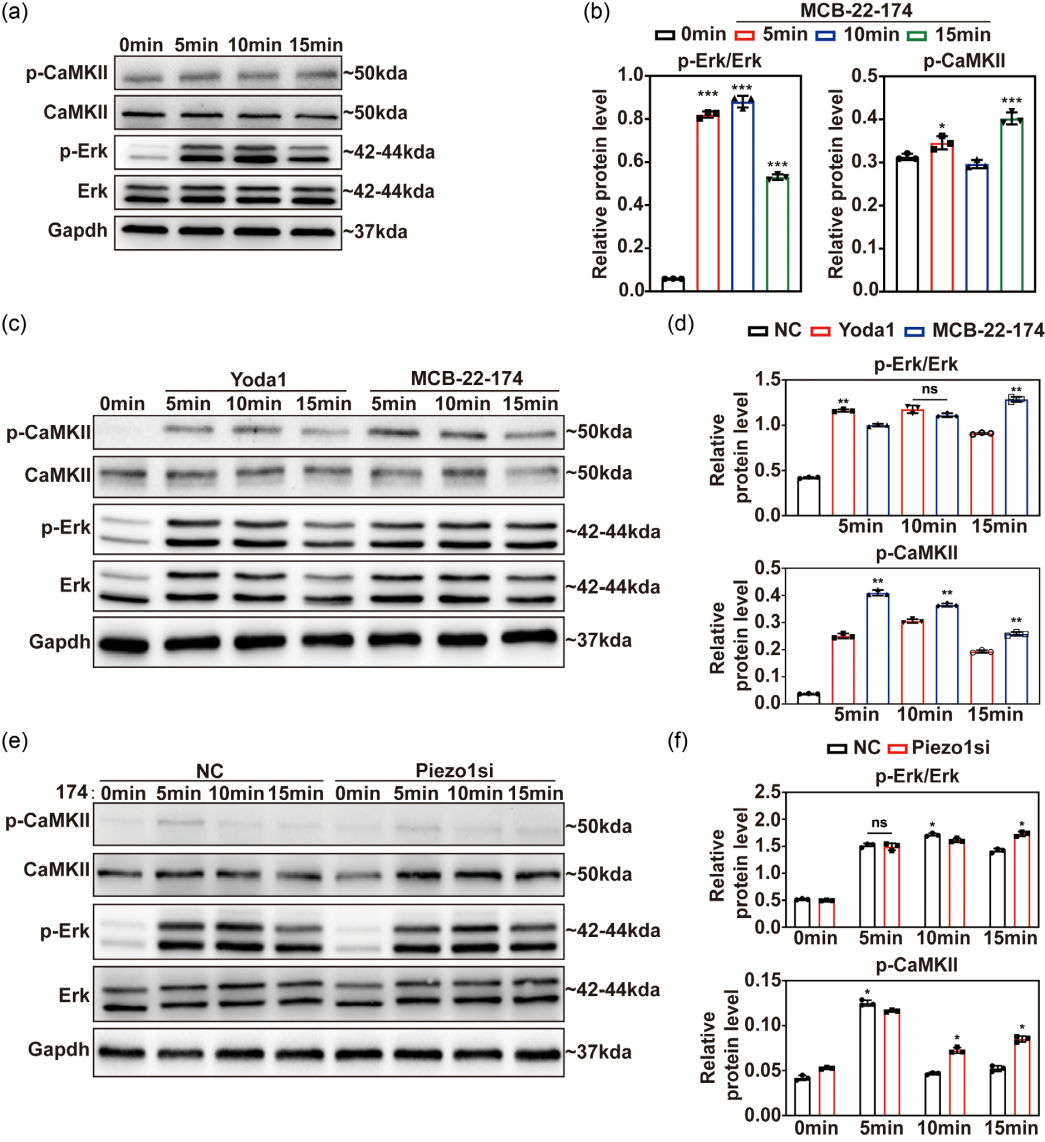
MCB‐22‐174 as a novel highly active agonist of Piezo1 could more effectively activate CaMKII/Erk signaling pathway than Yoda1 in vitro. a) Western blot images of CaMKII, p‐CaMKII, Erk, and p‐Erk in rMSCs after 0–15 min of 5 μmol L^−1^ MCB‐22‐174 treatment. b) Statistical data of p‐Erk/Erk ratio and p‐CaMKII relative expression. c) Western blot images of CaMKII, p‐CaMKII, Erk, and *p*‐Erk in rMSCs after 0–15 min treatment of 5 μmol L^−1^ Yoda1 or MCB‐22‐174. d) Statistical data of p‐Erk/Erk ratio and p‐CaMKII relative expression. e) Western blot images of CaMKII, p‐CaMKII, Erk, and p‐Erk in control and Piezo1 siRNA transfected rMSCs after 0–15 min of 5 μmol L^−1^ MCB‐22‐174 treatment. f) Statistical data of p‐Erk/Erk ratio and p‐CaMKII relative expression. For Western blot, GAPDH was chosen as internal reference (*n* = 3). *:*p* < 0.05, **:*p* < 0.01, ***p < 0.001.

As we have demonstrated that mechanical stimulation related to Piezo1 activation would further lead to ERK and CaMKII pathway activation, we further investigated whether nonmechanical chemical stimulation of MCB‐22‐174 would also activate these pathways. By treating C3H10T1/2 cell line with 5 μm MCB‐22‐174 for 0‐15 min, we found a remarkable upregulation of p‐ERK of 5 min after stimulation, along with upregulation of p‐CaMKII (**Figure** **4**a,b). This implies that activation of ERK and CaMKII signaling exhibited comparable outcomes with those brought by mechanical stimulation on MSCs. We also compared the pharmacological efficacy of Yoda1 and MCB‐22‐174 on the stimulated activation of Erk and CaMKII signaling pathways by treating rMSCs with 5 μmol L^−1^. Yoda1 or MCB‐22‐174 for 5–15 min. Results showed that MCB‐22‐174‐treated rMSCs generated more p‐CaMKII and a higher p‐Erk/Erk ratio, indicating a higher efficacy of MCB‐22‐174 (Figure 4c,d). To further clarify the specific activation of Piezo1 by MCB‐22‐174, we treated the siRNA knockdown rMSCs with MCB‐22‐174 for 0–15 min and tested ERK and CaMKII signaling pathways. We found a decline of p‐CaMKII and p‐ERK/ERK ratio in Piezo1 knockdown group after 5 min of application of MCB‐22‐174 (Figure 4e,f). Meanwhile, phosphorylations of ERK and CaMKII were increased in Piezo1 knockdown group at 10 and 15 min after MCB‐22‐174 application (Figure 4e,f). This phenomenon was probably generated by the delayed activation of Piezo1. Taken together, MCB‐22‐174 is a Piezo1‐specific agonist and can promote osteogenesis more effectively than Yoda1 in vitro.

### 2.5 MCB‐22‐174 Could Effectively Attenuate Disuse OP in the Hind‐Limb Unloading Model

**Figure 5:**
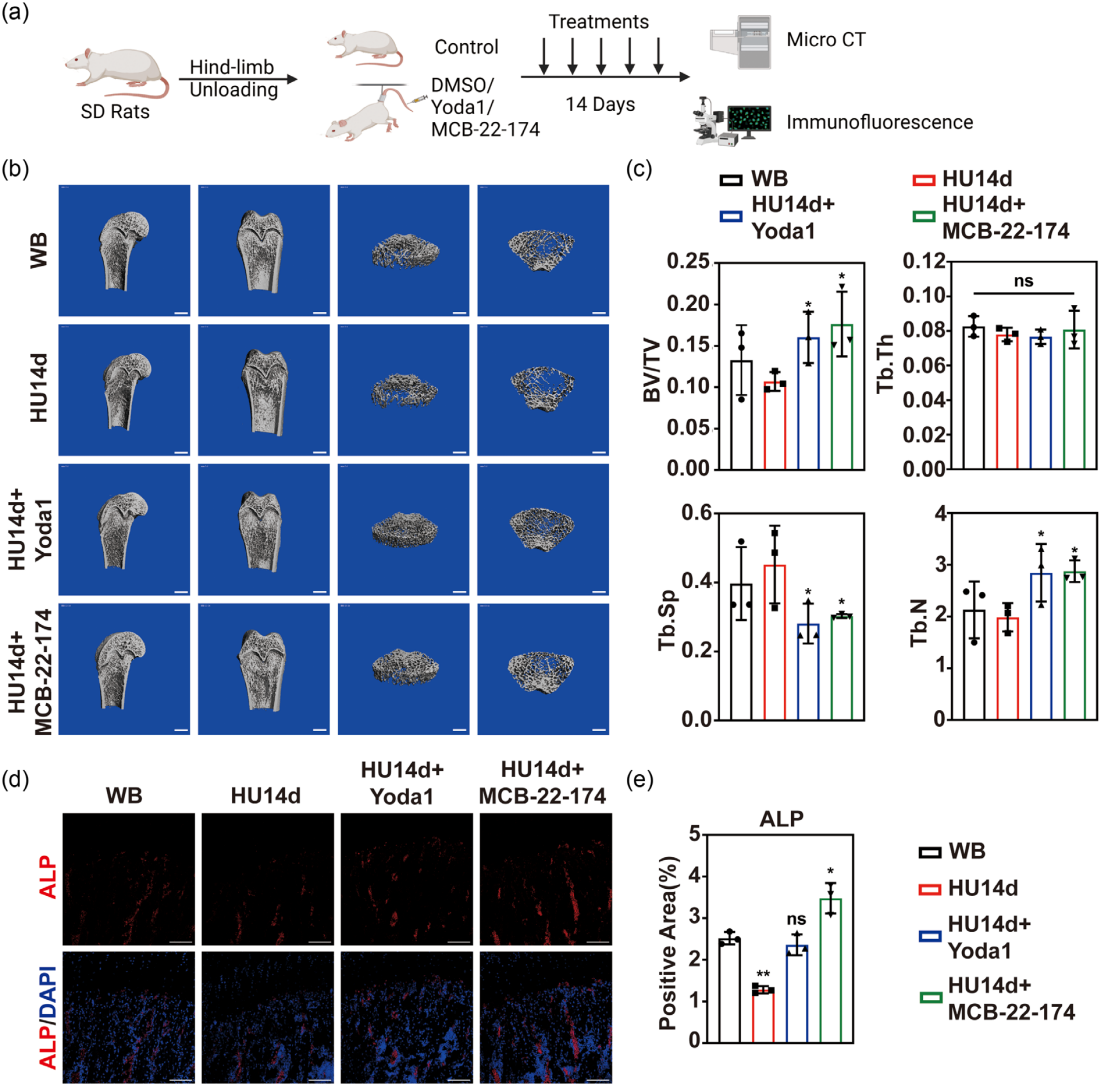
In vivo, MCB‐22‐174 could significantly improve bone quality and MSC osteogenesis during HU. a) The procedure of in vivo application of Yoda1 and MCB‐22‐174. Created with BioRender.com. b) μCT of rat right femur from control, HU group, HU group treated with 5 μmol kg^−1^ Yoda1, and HU group treated with 5 μmol kg^−1^ MCB‐22‐174. The left two lists of pictures are sagittal and coronal reconstruction images of the distal femur. The right two lists of pictures are the magnified trabecular bone structure from the most proximal growth plate of distal femur to the 200 slices underneath it. Scale bars equal to 2 mm. c) Statistic data of μCT (*n* = 3). d) Immunofluorescent staining of ALP in the proximal right tibia of the four groups. Nuclei were counterstained with DAPI. Scale bars equal to 100 μm. e) Statistical analysis of the fluorescent staining area (*n* = 3). ns. No significance *p < 0.05, **p < 0.01 comparing with WB group.

As we have developed MCB‐22‐174 as a reliable approach to activate Piezo1 and demonstrated its effect on in vitro osteogenesis of MSCs, we further attempted to apply MCB‐22‐174 on HU animal models, hopping to attenuate disuse OP effectively. We have already illustrated that during HU for 14 days, the total Piezo1 expression and activation in bone microenvironment was decreased with the loss of enough mechanical stimulation. Thus, we used MCB‐22‐174 or yoda1 to treat the disuse OP of the HU model by stimulating Piezo1 (**Figure** **5**a). In brief, we started HU at Day 0. Then HU rats were intraperitoneally injected with 5 μmol kg^−1^ MCB‐22‐174, Yoda1, or an equal amount of DMSO as control at Day 1,4,7,10, and 13. At Day 14, all rats were euthanized and μCT and immunofluorescence of bone formation markers were conducted (Figure 5a). The result of μCT demonstrated that the bone quality was improved after the application of MCB‐22‐174 or Yoda1, with upregulation of BV/TV and Tb.Th. and downregulation of Tb.Sp (Figure 5b,c).

We further investigated whether the novel mechanical stimulation mimicking agonist MCB‐22‐174 could improve bone morphology and promote osteogenesis in vivo. H–E staining of proximal tibia showed a larger trabecular bone area in MCB‐22‐174‐treated group compared to Yoda1‐treated group and DMSO control, indicating that MCB‐22‐174 treated group reached a better bone morphology in vivo (Figure S4, Supporting Information). In order to analyze the in vivo osteogenesis, we conducted immunofluorescent staining of alkaline phosphatase (ALP). We found a significant increase of ALP fluorescent area in MCB‐22‐174‐treated group, indicating a more active osteogenesis (Figure 5d,e). These results showed that MCB‐22‐174 is a reliable in vivo mechanical mimicking component that could attenuate disuse OP effectively in HU animal models.

## 3 Discussion

Mechanical forces are indispensable for bone homeostasis including skeletal formation, resorption, and adaptation. Loss of mechanical stimulation can therefore significantly weaken the bone structure, causing disuse OP and increasing the risk of fracture.^[^ ^22^ ^]^ Mechanosensitive ion channels, especially piezoelectric ion channels (Piezo1 and Piezo2), play important roles in the transduction of physical stimuli from a cell's surrounding environment and the translation of these stimuli into biochemical responses.^[^ ^9^, ^23^ ^]^ Piezo1 is mainly expressed in nonsensory tissues and plays an essential role in cardiovascular development, RBC cell volume, and bone formation.^[^ ^24^ ^]^ Piezo2 is mainly expressed in sensory tissues and serves as the principal mechanosensitive channel responsible for touch, proprioception, and baroreception.^[^ ^24^ ^]^ Previous studies have illustrated that Piezo1‐mediated mechanotransduction has been determined to be a key regulator of bone remodeling and homeostasis, regulating both osteogenic linage cells and osteoclasts in OP.^[^ ^10^, ^25^ ^]^ Thus, we further investigated whether Piezo1 could be a potential therapeutic target. In our work, we generated HU model, and found a significant decline of Piezo1 expression in HU14d group, indicating Piezo1 is an important regulator in disuse OP model. Piezo1 was demonstrated as a key force sensor in MSCs before, regulating MSCs proliferation and osteogenesis.^[^ ^10^, ^26^ ^]^ Using the CMS cell model, we further found a promoted MSCs proliferation and osteoblastic differentiation, while these processes could be interfered by knockdown of Piezo1, thus validating the necessity of Piezo1 in MSCs’ mechanical sensing. These results illustrated the significant priority of Piezo1 in MSCs’ mechanical stimulation sensing and disuse OP and ensured the therapeutic feasibility of targeting Piezo1.

In 2015, Syeda et al. first developed a small molecule called Yoda1 as a chemical approach of Piezo1 activation,^[^ ^12^ ^]^ highlighting the accessibility of activating Piezo1 by chemical molecules. Importantly, not only Yoda1 was reported as a Piezo1 activity modulator,^[^ ^27^ ^]^ Jedi1 and Jedi2 which are structurally different from Yoda1 are other small molecules that pharmacologically trigger Piezo1 activation.^[^ ^28^ ^]^ However, the EC_50_ of Jedi1 and Jedi2 is ≈200 and 158 μmol L^−1^, which is much less potent (i.e., “no better”) than Yoda1, whose estimated EC_50_ is ≈17.1 μmol L^−1^.^[^ ^12^, ^28^ ^]^ Based on these, modification of the chemical structure of Yoda1 with the aim of increasing its ability to modulate Piezo1 is urgently needed. Parsonage G et.al. generated KC159 and KC289, 4‐benzoic acid‐modified compounds of Yoda1, as novel Piezo1 agonist, with approximately threefold increase over the EC_50_ of Yoda1.^[^ ^29^ ^]^ In our work, we modified Yoda1 and developed MCB‐22‐174, which could neatly fit the pocket structure of Piezo1 and thus present an eightfold higher potency over the EC_50_ of Yoda1. Further, we found the cell viabilities of MSCs still reached over 80% at 16 μmol L^−1^ MCB‐22‐174 treatment for 2 days, indicating an ensured safety of its application. Previous studies have demonstrated Piezo1 agonist, Yoda1, could promote MSC osteoblastic differentiation.^[^ ^26^, ^27^ ^]^ In our study, we observed a better osteogenesis property by treating C3H10T1/2 cell line with MCB‐22‐174 than Yoda1, due to its enhanced efficacy on Piezo1 activation. Together, we demonstrated that MCB‐22‐174 is a novel Piezo1 agonist with high efficacy and ensured safety, which have potential clinical application for the treatment of disuse OP.

Piezo1 activation could promote MSC proliferation and osteoblastic differentiation, and some efforts were made to reveal the underlying mechanism. Hu et al. emphasized the critical role of Wnt/β‐catenin/ATF4 in MSCs stemness after Piezo1 activation.^[^ ^26^ ^]^ Zhou et al. found Piezo1‐activated Ca2+ influx could stimulate Calcineurin, which promotes concerted activation of NFATc1, YAP1, and ß‐catenin transcription factors by inducing their dephosphorylation as well as NFAT/YAP1/ß‐catenin complex formation.^[^ ^10^ ^]^ CaMKII signaling pathway regulates transcription and cell proliferation.^[^ ^30^ ^]^ MAPK‐ERK signaling pathway is one of the most important for cell proliferation.^[^ ^18^ ^]^ However, the interaction between Piezo1 and CaMKII/Erk axis and the outcomes involved in the regulation of MSCs proliferation have not been investigated. In our work, we demonstrated that either CMS or Yoda1‐activated Piezo1 could activate the CaMKII/Erk axis in MSCs, whereas the Erk activation is attenuated by knockdown of Piezo1. Further, we found that CMS‐promoted MSC proliferation could be inhibited by antagonists of CaMKII and Erk, that indicated the significance of CaMKII/Erk axis in MSC proliferation. In addition, we validated the effect of MCB‐22‐174 in Piezo1/CaMKII/Erk axis in MSCs and found a stronger activation of CaMKII/Erk signaling relative to Yoda1. Together, these data suggested MCB‐22‐174 can promote MSC proliferation via Piezo1/CaMKII/Erk axis.

Disuse OP occurs in varied scenarios and currently approved therapies for disuse OP are mainly including restoring of mechanical stress and antiresorption drugs such as bisphosphonates and denosumab or bone anabolic drugs. In cosmonauts after space flight, the tibial cortical density recovered, but the cortical porosity and trabecular bone did not fully recover even after 1 year postlanding.^[^ ^29^ ^]^ In disuse OP, the trabecular bone recovered to baseline after 6 weeks of weight bearing (WB), but the bone mineral density continued to decrease after continuous 13‐week WB.^[^ ^31^ ^]^ The treatment of disuse OP by combining antiresorption drugs such as denosumab or bone anabolic drugs with physical exercise has shown sometimes inconsistent results.^[^ ^1^ ^]^ Moreover, Li et al.^[^ ^32^ ^]^ pointed out that long‐term disuse OP showed less sensitivity to bisphosphonates. These indicated an urgent need of disease‐specific therapeutic strategies for disuse OP. In our work, we further attempted to applicate MCB‐22‐174 in vivo to treat disuse OP in HU rodent model. We found that MCB‐22‐174 showed greater osteogenic promotion ability than that of Yoda1 and attenuated OP successfully in the HU model in vivo, indicating that MCB‐22‐174 is probably a drug‐like small molecule to treat disuse OP. For OP in normal WB conditions, Sun et al.^[^ ^11^ ^]^ reported a decline of Piezo1 expression in the bone specimens from OP patients, while Guan et al.^[^ ^33^ ^]^ developed a bone‐targeting nanocarrier with effects of Piezo1 activation that could effectively improve osteoporotic bone repair. To this point, the MCB‐22‐174 may be a promising Piezo1 agonist for treating OP in WB bones.

Our work introduced MCB‐22‐174 a novel high activity agonist of Piezo1, which is promising in disuse OP treatment by promoting MSCs osteoblastic differentiation and proliferation via activating Piezo1/CaMKII/Erk signaling pathway. Moreover, bone remodeling tightly relies on the cooperation of osteoclasts and osteoblasts together with osteocytes, bone lining cells, osteomacs, and endothelial cells in the bone microenvironment within the basic multicellular unit (BMU).^[^ ^34^ ^]^ In this research, we demonstrated the osteogenic function of MCB‐22‐174 and its mechanism on MSCs. The function of Piezo1 and MCB‐22‐174 on other cell types in BMU is a fascinating work to further clarify the role of Piezo1 in bone remodeling and develop novel targeting strategies for disuse OP treatment.

Since Piezo1 is expressed not only in bone‐related cells but also in other cell types,^[^ ^23^ ^]^ its effects on other organs and bone metabolism via other organs should be further explored. In vascular endothelial cells, Piezo1 activation is related to vascular calcification and inflammation release.^[^ ^35^, ^36^ ^]^ Thus, tissue‐specific targeting activation of Piezo1 by the novel agonist MCB‐22‐174 could potentially be a better strategy for the precise treatment of diseases.

## 4 Experimental Section

4.1

4.1.1

##### Rats and Pharmacological Treatment

Standard deviation (SD) rats were purchased from Shanghai Jihui Experimental Animal Breeding Co., Ltd. through the experimental animal center of Xinhua Hospital affiliated with Shanghai Jiao Tong University School of Medicine. Two‐month‐old rats were subjected to HU and euthanized after 14 days. The procedure of HU models was described in the previous study.^[^ ^37^ ^]^ In brief, the tails of rats were suspended and fixed on the upper lids of cages, and the hind limbs were unloaded, but forelimbs were unaffected (Figure S1a, Supporting Information). Sufficient food and water were placed within the reach of the HU rats. Control groups were set at a normal WB condition. 5 μmol kg^−1^ of Yoda1 and MCB‐22‐174 were injected on 1, 4, 7, 10, and 13 days after HU intraperitoneally at a concentration of 1 mmol L^−1^. All of the rats were maintained in a specific pathogen‐free animal facility of the experimental animal center (the Xinhua Hospital affiliated with Shanghai Jiao Tong University School of Medicine.). The animal experiments were approved by the Ethics Committee of Xinhua Hospital Afiliated to Shanghai Jiaotong University School of Medicine (XHEC‐NSFC‐2023‐318). The right femurs of the rats were scanned with Scanco Medical CT‐40 instruments (Scanco Medical AG, Bruttisellen, Switzerland) at a resolution of 8 μm. The trabecular bone of the distal femur was structurally analyzed from the most proximal femur growth plate to the 200 slices underneath it.

##### Immunofluorescence

The right tibias of rats were isolated and fixed in 4% paraformaldehyde for 24 h, decalcified in 12.5% EDTA (pH 7.0) for 10 weeks, and embedded in paraffin. Longitudinal sections with a thickness of 8 μm were cut for immunofluorescence. We used Piezo1 (1:100, Abclonal, Wuhan, China), ALP (1:100, Abclonal), and Lepr (1:100, Abclonal) as immunofluorescent‐staining first antibodies. Antigen retrieval used citrate antigen retrieval solution (pH = 6.0) (MX Biotech, China) at 65 °C overnight and 5% bull serum albumin for blocking. Sections were incubated with antibodies overnight at 4 °C. Alexa Fluor 647 AffiniPure Rabbit Anti‐Goat IgG(H + L) (1:200, Yeason Biotech, China) was used as a secondary antibody. Fluorescent secondary antibody incubation of sections was conducted for 1 h at room temperature from light. For immunofluorescent costaining, we used the multicolor immunofluorescent staining kit (abs50029, absin, China) following the manufacturers’ instruction. Sections after immunofluorescent staining were then counterstained with DAPI (1:1000, Yeason Biotech) and sealed with an antifade mounting medium (Beyotime Biotech, China). The images were collected through confocal microscopy (Leica, Buffalo Grove, IL, USA). Statistical analysis of the percentage of light area (pixels) was conducted by ImageJ. All of the parameters were calculated based on the standardized bone histomorphology.

##### Cell Culture

MSCs were isolated from 4 week‐old male mice and rats. Bone marrow cells were flushed out from the tibias and femurs and cultured in DMEM containing 10% fetal bovine serum (FBS) and 1% penicillin–streptomycin for 48 h at 37 °C incubator with 5% CO_2_. Nonadherent cells were washed and abolished, and then MSC was isolated. The characteristics of MSC were confirmed based on previous laboratory reports,^[^ ^38^ ^]^ and passage 3–5 was used for experiments. C3H10T1/2 cell linage was purchased from the National Collection of Authenticated Cell Cultures (China), and the ones between passages 15‐30 were used for experiments. All cells were cultured at 37 °C incubator with 5% CO_2_ and DMEM containing 10% FBS and 1% penicillin–streptomycin for incubation and experiment.

##### RT‐qPCR Analysis

Total RNA from cultured cells and bone was isolated with an RNA isolator (Vazyme, Nanjing, China). cDNAs were synthesized using a reverse transcription kit (Takara, Kusatsu, Japan), and RT‐qPCR was performed in a ViiA 7 real‐time polymerase chain reaction (PCR) standard deviation system (Applied Biosystems) using SYBR GREEN (Yeason Biotech). GAPDH was used as an internal control for cDNA. The primers used in this study are described in Table S1, Supporting Information.

##### Western Blot Analysis

Cultured cells were lysed in RIPA buffer (50 mm Tris‐HCl, pH 7.4, 1% Nonidet P‐40, 150 mm NaCl, and 0.1% SDS) and protease inhibitors were added (10 mg mL^−1^ leupeptin, 10 mg mL^−1^ pepstatin A, and 10 mg mL^−1^ aprotinin) for 15 min on ice. Protein fractions were centrifuged at 12 000 rpm at 4 °C for 15 min. Then, the supernatants were collected for concentration detection and boiled with 4× loading buffer (Takara) for 10 min. Subsequently, the boiled protein solutions were subjected to SDS‐PAGE and transferred to prepared NC membranes. The transferred membranes were blocked with 5% BSA‐TBST solution and incubated with antibodies at 4 °C overnight. The primary antibodies we used are listed as follows: p‐Erk (Cell Signaling Technology, 4370, 1:1000), Erk (Cell Signaling Technology, 4695, 1:1000), p‐CaMKII (Cell Signaling Technology, 12 716, 1:1000), CaMKII (Cell Signaling Technology, 4436, 1:1000), and Piezo1 (Abclonal, A23380, 1:1000). HRP‐conjugated secondary antibodies (Cell Signaling Technology) were incubated at 1:2000 dilutions. The antibody complexes were visualized using an enhanced chemiluminescence detection system (Tanon, China).

##### Piezo1 Small‐Interfering RNA (siRNA) Transfection

SiRNA was purchased from Shanghai Genepharma Biotechnology. 2OD siRNA was initially dissolved in 125 μL DEPC water for subsequent use. For siRNA transfection in one well of a 6‐well plate, 2.5 μL Lipofectamine 2000 and 5 μL siRNA solution were used. Negative control RNA sections were employed in control groups. The transfection was performed in a cell incubator for 5 h and later the medium was with DMEM containing 10% FBS and 1% penicillin–streptomycin. Quantitive real‐time PCR  was performed after 2 days to identify the knockdown of Piezo1(Figure S1b, Supporting Information). The siRNA sequences are shown in Table S2, Supporting Information.

##### In Vitro Piezo1 Agonist Treatment

Piezo1 agonist, Yoda1, was purchased from MedChemExpress (HY‐18 723). We generated MCB‐22‐174 at Shanghai Jiao Tong University School of Pharmacy (Figure S2a–c, Supporting Information). All these drugs were diluted to 20 mm by DMSO and used to treat cells at a final concentration of 3 or 5 μm in a culture medium as described in Results.

##### Cyclic Mechanical Stretch

FX‐5000T Flexcell Tension Plus system (Flexcell International Corporation, Hillsborough, NC, USA) was applied to provide CMS. rMSCs were cultured on 6‐well BIOFLEX culture plates (Flexcell International Corporation, Hillsborough, NC, USA) with flexible membrane bottoms at 2 × 10^5^ cells well^−1^. CMS was applied to the stretch group in a sinusoidal waveform with a 10% amplitude at 0.5 Hz as previously reported.^[^ ^39^ ^]^ The control cells were cultured in similar static plates and incubated in the same incubator.

##### Flow Cytometry and EdU Proliferation Analysis

BeyoClick EdU Cell Proliferation Kit (Beyotime, Shanghai, China) was used for EdU proliferation analysis. The protocol was followed based on the manufacturer's instructions. In brief, MSCs of rats differently treated were incubated with EdU working solution for 2 h before collection. After that the collected cells (around 2 × 10^6^) were fixed, treated with 0.3% Triton‐X buffer, and washed with phosphate buffer saline (PBS) (2% FBS). Then the samples were incubated with Click Reaction Buffer for 30 min in a dark room following the instructions and were washed with PBS (2% FBS) three times. Then cell suspensions were subjected to flow cytometry analyses using a Guava easyCyte flow cytometer (Luminex, Germany). The gates were set according to the manufacturer's instructions. All the samples in the same experiments and analysis were gated under the same parameters by FlowJo.

##### Calcium Influx Detection

Calbryte 520 AM (AAT bioquest, US) was used to detect the influx of calcium ions following the manufacturer's instruction. In brief, C3H10T1/2 cell line was cultured in 96‐well plates (2 × 10^3^ cells well^−1^) for 1 day; then we replaced the culture medium with 100 μL dye working solution and drug treatment (PBS added 2 μmol L^−1^ Calbryte 520 AM, 10% FBS, and Yoda1 or MCB‐22‐174 at different concentration levels) for 2 h. The solution was replaced with PBS and measured at fluorescent intensity of Ex/Em = 490/525 nm.

##### Statistical Analysis

Statistical data graphs were made with Graphpad Prism 8 (Boston, MA). A two‐tailed student's *t*‐test was used for comparison between the two groups. *P* < 0.05 was considered statistically significant. At least three independent replicates were used for each experiment. The results were expressed as mean ± SD.

## Conflict of Interest

The authors declare no conflict of interest.

## Supporting information

Supplementary Material

## Data Availability

The data that support the findings of this study are available from the corresponding author upon reasonable request.
