# Supplementary material for: A Novel Piezo1 Agonist Promoting Mesenchymal Stem Cell Proliferation and Osteogenesis to Attenuate Disuse Osteoporosis
Source: Small Sci. 2024 Jun 30;4(9):2400061. doi: 10.1002/smsc.202400061 (PMC11935128; doi:10.1002/smsc.202400061)
Supplement: Supplementary file 1 — Supplementary Material [file SMSC-4-2400061-s001.pdf]

Supporting Information

A Novel Piezo1 Agonist Promotes Mesenchymal Stem Cell Proliferation and Osteogenesis to Attenuate Disuse Osteoporosis

Authors

Ruihan Hao[a,#], Hairong Tang[b,#], Chunyong Ding[b], Rajbanshi Bhavana[c], Yuhang Liu[a], Ding Ma[a], Zhouyi Duan[a], Yuxin Qi[d], Liming Dai[a], Bingjun Zhang[a,*], Ao Zhang[b,*], Xiaoling Zhang[a,*]

Affiliations

a. Department of Orthopedic Surgery, Xin Hua Hospital Affiliated to Shanghai Jiao Tong University School of Medicine (SJTUSM), Shanghai 200092, China

b. Shanghai Frontiers Science Center of Targeted Drugs, School of Pharmaceutical Sciences, Shanghai Jiao Tong University, Shanghai 200240, China

c. Department of Dermatology and Venereology, Tongji University School of Medicine, Shanghai 200092, China

d. Collaborative Innovation Centre of Regenerative Medicine and Medical BioResource Development and Application Co-constructed by the Province and Ministry, Guangxi Medical University, Nanning, Guangxi 530021, China.

[#] Ruihan Hao and Hairong Tang are the co-first authors and contributed equally to the whole work.

*Corresponding author: Xiaoling Zhang

E-mail: xlzhang@shsmu.edu.cn(X.Zhang)

Co-corresponding author: Ao Zhang

E-mail: ao6919zhang@sjtu.edu.cn

Co-corresponding author: Bingjun Zhang

E-mail: smior122@163.com (B. Zhang)

Keywords: Disuse Osteoporosis; Piezo1; Novel Agonist; MSC proliferation; Osteogenesis

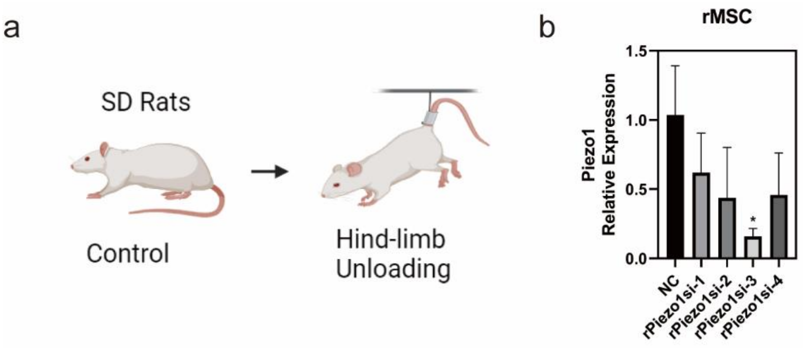

Figure S1. Rat experiment methods and Piezo1 siRNA validation.

a. The sketch illustrator of hind-limb unloading. Created with BioRender.com. b. Relative Piezo1 mRNA expression in control and rPiezo1 siRNA 1-4 treated group. *, $p < 0.05$ comparing with NC group.

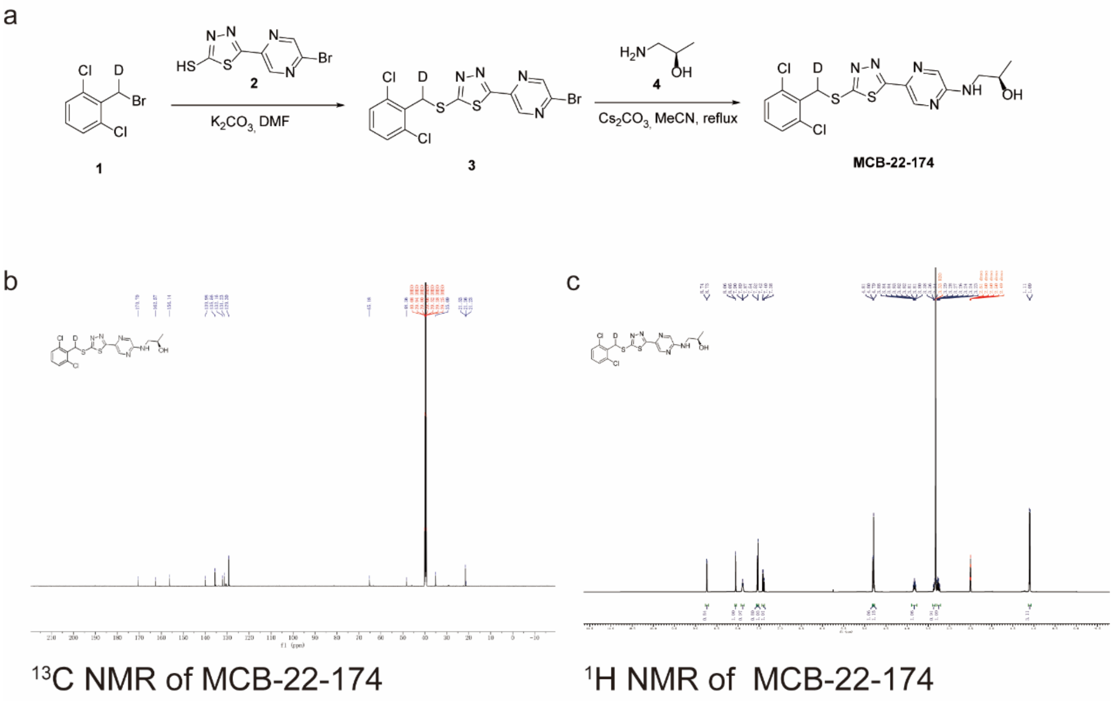

Figure S2. Synthesis and identification of MCB-22-174

a. A 25 mL round-bottom flask was charged with 1 (200 mg, 0.83 mmol), 2 (228 mg, 0.83 mmol), potassium carbonate (229 mg, 1.66 mmol), and N,N-dimethylformamide (DMF, 4 mL), and the resulting solution was stirred for 6 h at room temperature. Water (30 mL) was added into the solution, and the mixture was extracted with ethyl acetate (20 mL) for 2 times. The organic layer was combined, washed with brine, dried over anhydrous Na_2SO_4 and filtered. The solvent was removed under reduced pressure and the residue was purified by flash column chromatography (elute with hexane/ethyl acetate = 10:1, v/v) to generate intermediate **3**. Yellow solid (274 mg), yield 76%. ^{1}H NMR (400 MHz, $CDCl_3$): δ 9.31 (d, J = 1.4 Hz, 1H), 8.58 (d, J = 1.4 Hz, 1H), 7.31 (d, J = 8.1 Hz, 1H), 7.23 (d, J = 8.2 Hz, 1H), 7.19-7.15 (m, 1H), 5.00 (s, 1H). ESI-MS (m/z): 436.16 (M + H^+). A 25 mL round-bottom flask was charged with **3** (100 mg, 0.23 mmol), **4** (34.5 mg, 0.46 mmol), cesium carbonate (149.8 mg, 0.46 mmol), and

Acetonitrile (MeCN, 4 mL), and the resulting solution was stirred for 2 h at 82 °C. Water (20 mL) was added into the solution, and the mixture was extracted with ethyl acetate (15 mL) for 2 times. The organic layer was combined, washed with brine, dried over anhydrous Na_2SO_4 and filtered. The solvent was removed under reduced pressure and the residue was purified by flash column chromatography (elute with dichloromethane/methanol = 20:1, v/v) to generate compound MCB-22-174. White solid (78.9 mg), yield 80%. ^{13}C NMR (150 MHz, DMSO-d_6): δ 170.7, 162.6, 156.1, 139.9, 135.6, 132.2, 131.2, 129.3, 65.2, 48.4, 35.1, 21.5. ^{1}H NMR (400 MHz, DMSO-d_6): δ 8.73 (d, J = 1.4 Hz, 1H), 8.05 (d, J = 1.4 Hz, 1H), 7.89 (t, J = 6.0 Hz, 1H), 7.54 (s, 1H), 7.52 (s, 1H), 7.42 - 7.38 (m, 1H), 4.81 (d, J = 4.8 Hz, 1H), 4.79 (s, 1H), 3.85 - 3.80 (m, 1H), 3.38 - 3.34 (m, 1H), 3.29 - 3.23 (m, 1H), 1.10 (d, J = 6.2 Hz, 3H). ESI-MS (m/z): 430.32 ($M + H^+$).

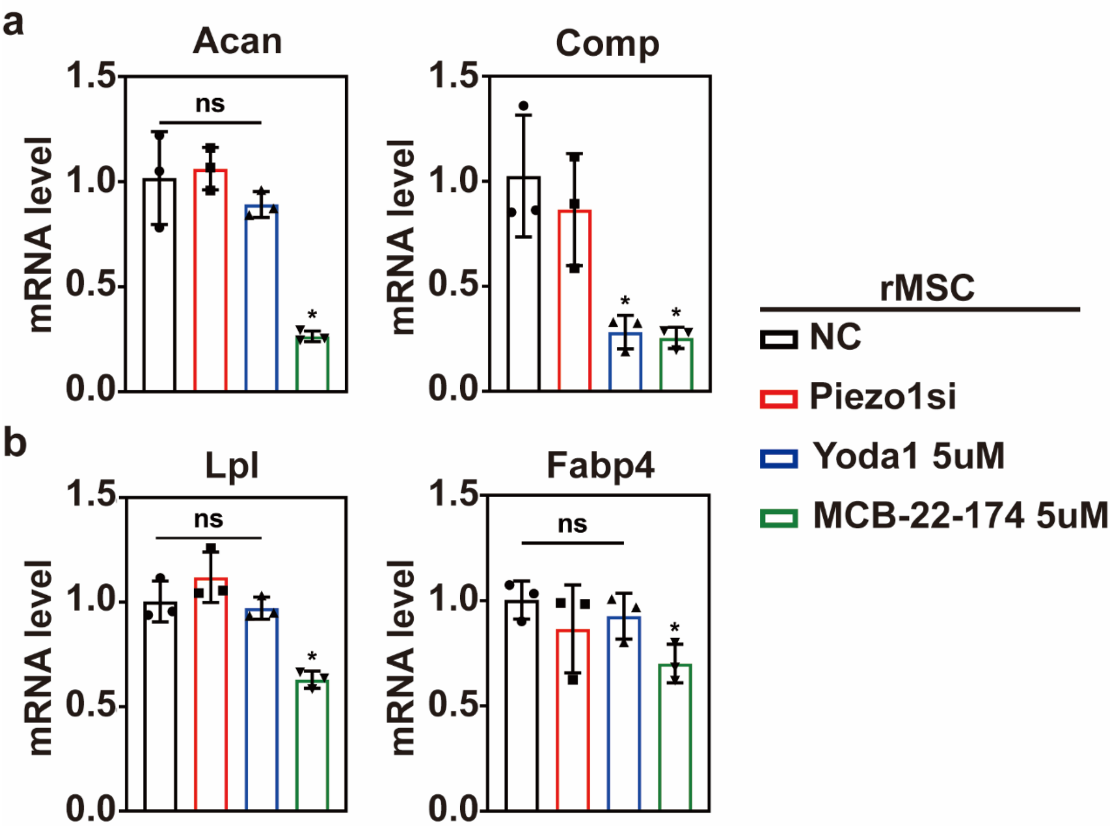

Figure S3. MCB-22-174 suppressed chondrogenesis and adipogenesis.

a. Relative expression of Acan and Comp in control and Piezo1siRNA, 5 μmol·L-1 Yoda1 or 5 μmol·L-1 MCB-22-174 treated groups (n=3). b. Relative expression of Lpl and Fabp4 in control and Piezo1siRNA, 5 μmol·L-1 Yoda1 or 5 μmol·L-1 MCB-22-174 treated groups (n=3). ns, no significance; *, $p < 0.05$ comparing with NC group.

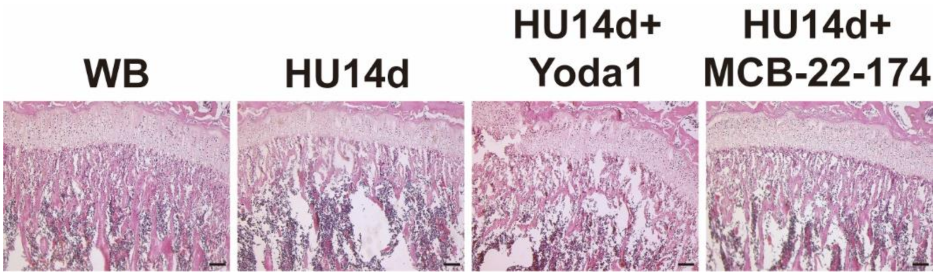

Figure S4. MCB-22-174 improved in vivo trabecular bone morphology.
Representative images of H-E staining of all 4 groups. Scale bars equal to 100μm.

Supplementary Table S1. The sequence of primers used for RT-qPCR analysis.

Gene names	Forward primer	Reverse primer
mouse-Col1a1	GGGGCAAGACAGTCATCGAA	GAGGGAACCAGATTGGGGTG
mouse-Runx2	GACTGTGGTTACCGTCATGGC	ACTTGGTTTTTCATAACAGCGGA
mouse-Sp7 (Osx)	ACCCCAAGATGTCTATAAGCCC	CGCTCTAGCTCCTGACAGTTG
mouse-Gapdh	AGGTCGGTGTGAACGGATTTG	TGTAGACCATGTAGTTGAGGTCA
rat-Piezo1	ACGCTTCAATGCTCTCTCGT	ACGTTTGCCCAAAGGTTACA
rat-Col1a1	TGGTACATCAGCCCAAACCC	CAGGATCGGAACCTTCGCTT
rat-Runx2	GCCTTCAAGGTTGTAGCCT	TGAACCTGGCCACTTGGTTT
rat-Sp7 (Osx)	GCCAGTAATCTTCGTGCCAG	GGACTGGAGCCATAGTGAGC
rat-Gapdh	GCTGAGAATGGGAAGCTGGT	CTCGTGGTTCACACCCATCA
rat-Col2a1	AGGATGTATGGAAGCCCTCGT	GGCCCTAATTTTCCACTGGC
rat-Acan	GACACCCCTACCCTTGCTTC	GGTCGATCTCACACAGGTCC
rat-Comp	CCACTGCCTGCGTTCTAGTG	CATTCCGCAAGCGTCACATT
rat-Cebpa	AAGTGTCCCCACCCCTAGTT	CCCTTCTCCACGAACTCACC
rat-Adipoq	CTGGCTCCAAGTGTATGGGG	TTTGATTCTCGGGGCTACGG
rat-Lpl	GTTGCGTGCAAAGTGAGGAG	AACAAAAGAGCGGCAACGAG

rat-Fabp4	TCGTCATCCGGTCAGAGAGT	TCATGACACATTCCACCACCA

Supplementary Table S2. The sequence of siRNA of rat Piezo1.

	sense (5'-3')	antisense (5'-3')
rPiezo1si-1	GGAGGAGGAUGACAUAGAUTT	AUCUAUGUCAUCCUCCUCCTT
rPiezo1si-2	CACGAGUACUCCAGUAACUTT	AGUUACUGGAGUACUCGUGTT
rPiezo1si-3	GGUCCUAUCUGGAUAUGCUTT	AGCAUAUCCAGAUAGGACCTT
rPiezo1si-4	GUGUCUUCGUGGAGCAAAUTT	AUUUGCUCCACGAAGACACTT

rPiezo1si-3 was used for further experiments.
